# An attenuated Machupo virus with a disrupted L-segment intergenic region protects guinea pigs against lethal Guanarito virus infection

**DOI:** 10.1038/s41598-017-04889-x

**Published:** 2017-07-05

**Authors:** Joseph W. Golden, Brett Beitzel, Jason T. Ladner, Eric M. Mucker, Steven A. Kwilas, Gustavo Palacios, Jay W. Hooper

**Affiliations:** 1 Department of Molecular Virology, Virology Division, United States Army Medical Research Institute of Infectious Diseases, Fort Detrick, MD 21702 United States; 2 Center for Genome Sciences, United States Army Medical Research Institute of Infectious Diseases, Fort Detrick, MD 21702 United States

## Abstract

Machupo virus (MACV) is a New World (NW) arenavirus and causative agent of Bolivian hemorrhagic fever (HF). Here, we identified a variant of MACV strain Carvallo termed Car^91^ that was attenuated in guinea pigs. Infection of guinea pigs with an earlier passage of Carvallo, termed Car^68^, resulted in a lethal disease with a 63% mortality rate. Sequencing analysis revealed that compared to Car^68^, Car^91^ had a 35 nucleotide (nt) deletion and a point mutation within the L-segment intergenic region (IGR), and three silent changes in the polymerase gene that did not impact amino acid coding. No changes were found on the S-segment. Because it was apathogenic, we determined if Car^91^ could protect guinea pigs against Guanarito virus (GTOV), a distantly related NW arenavirus. While naïve animals succumbed to GTOV infection, 88% of the Car^91^-exposed guinea pigs were protected. These findings indicate that attenuated MACV vaccines can provide heterologous protection against NW arenaviruses. The disruption in the L-segment IGR, including a single point mutant and 35 nt partial deletion, were the only major variance detected between virulent and avirulent isolates, implicating its role in attenuation. Overall, our data support the development of live-attenuated arenaviruses as broadly protective pan-arenavirus vaccines.

## Introduction

Members of the *Arenaviridae* are enveloped ambisense single-stranded RNA viruses with two segments, small (S) and large (L), encoding a 10.7 Kb genome expressing five distinct proteins^1–3^. The L-segment encodes the matrix ring finger Z protein^4^ and the polymerase L protein (L-segment)^5^. The S-segment encodes the nucleoprotein (NP) and the glycoprotein precursor (GPC)^6^. GPC is cleaved into two glycoproteins, GP1 and GP2 by the cellular protease S1P^7^. Each RNA segment encodes two ORFs and also contains noncoding regions including 5′ and 3′ untranslated regions (UTRs) and a non-coding intergenic region (IGR)^3, 8, 9^. Mammalian arenaviruses (genus *mammarenavirus*) are divided into the Old World (OW) and New World (NW) complexes based on geographical distribution and serology^2^. Machupo virus (MACV) is a member of the NW complex of arenaviruses and is the causative agent of Bolivian hemorrhagic fever (HF)^2^. Human infections generally result from exposure to chronically infected rodents (*Calomys callosus)* ^10^, but human-to-human spread has been reported^11^. Bolivian HF is a febrile illness often associated with vascular leakage and occasional concomitant neurological manifestations^11, 12^. Infection can result in a systemic inflammatory response syndrome leading to multiple organ failure and death. Other NW arenaviruses, including Junin virus (JUNV) and Guanarito virus (GTOV), also cause HF in South America^2, 13^. More recently other NW arenaviruses capable of causing human disease have emerged, including Sabia virus, Chapare virus and Whitewater Arroyo virus^2, 13–16^. Therefore, this family of viruses is an important group of emerging and re-emerging human pathogens.

No FDA licensed countermeasures exist to treat NW arenaviruses. Active and passive vaccine strategies, as well as small molecule inhibitors have been shown to be effective at reducing lethality in humans^17–19^. An attenuated live-virus vaccine, termed Candid#1, is currently used in populations at high risk to JUNV infection in Argentina^20–23^. Implementation of this vaccine in endemic regions reduced fatality rates substantially. Some evidence based on studies in guinea pigs and non-human primates (NHPs) suggest that Candid#1 can cross-protect against MACV^24^; however, these findings have not been validated in humans. Candid#1 was produced by passage of the virulent XJ strain twice in guinea pigs, 44 times in mouse brains and finally several passages in fetal rhesus lung diploid cells (FRhL-2)^23, 25–27^. This process produced a strain attenuated in humans, non-human primates, guinea pigs, and mice that also lacked neurotropism in animal models. The exact nature of the attenuation is enigmatic and recent evidence suggests that a single amino acid change in the GP2 transmembrane region restores virulence in neonatal mice^25^. Other NW arenavirus vaccine strategies have included the use of Tacaribe virus (TACV), an arenavirus serologically related to JUNV and MACV believed to be apathogenic in humans. In animal models, TACV functions as a vaccine against JUNV^28, 29^. However the underlying mechanism(s) attenuating TACV in humans is unclear. Glycoprotein-targeting subunit vaccines based on modified vaccine Ankara or Venezuelan equine encephalitis replicon vectored systems protect against lethal infection by JUNV in guinea pig models^30, 31^. However, because of heterogeneity in the glycoproteins^6, 28, 32^, it is unlikely these vaccines will provide sufficient cross-protection against heterologous arenaviruses. Thus, alternative strategies aimed at producing safe and broadly protective arenavirus vaccines are needed.

MACV strain Carvallo is the prototypical MACV strain first isolated in 1963^33, 34^. Several studies report that strain Carvallo is pathogenic in guinea pigs, with lethality upwards of 60%^28, 35^. We previously reported that a MACV strain Carvallo variant (Car^91^) does not cause severe disease in guinea pigs^36^. Here, we found an earlier passage of strain Carvallo (Car^68^) was virulent in guinea pigs. Subsequently, we identified genomic differences between virulent (Car^68^) and avirulent (Car^91^) Carvallo strains. Additionally, because Car^91^ was apathogenic in guinea pigs, but produced detectable humoral immune responses, we investigated if it could function as a live-attenuated vaccine and protect against more distantly related NW arenaviruses.

## Results

### MACV strain Car^68^ and Chic are lethal in guinea pigs, while strain Car^91^ is attenuated

**Figure 1:**
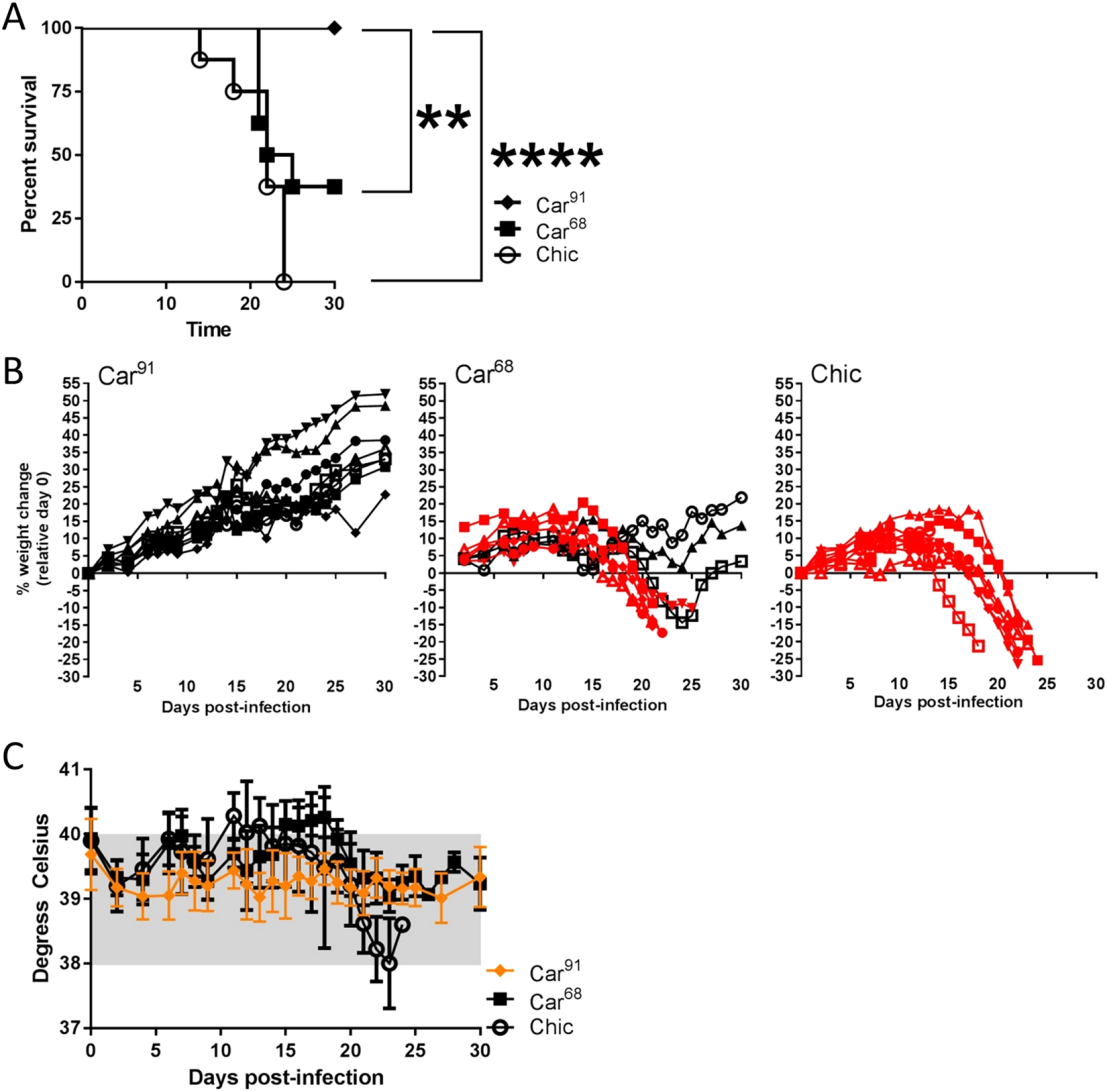
Infection of Hartley guinea pigs with MACV strain Car^68^, Car^91^ and Chicava. (**A**) Guinea pigs were challenged with 1,000 pfu of Car^91^, Car^68^ and Chic by the i.p. route. Survival was monitored for 30 days post-infection. Asterisks denote statistical significance. (**B**) Percent weight loss for individual guinea pigs in the indicated groups was plotted based on day 0 starting weight. Animals succumbing to infection are shown in red. (**C**) The mean group temperatures were plotted. The normal temperature range is shaded in grey. To aid visualization, data for Car^91^-infected animals is depicted in orange.

**Figure 2:**
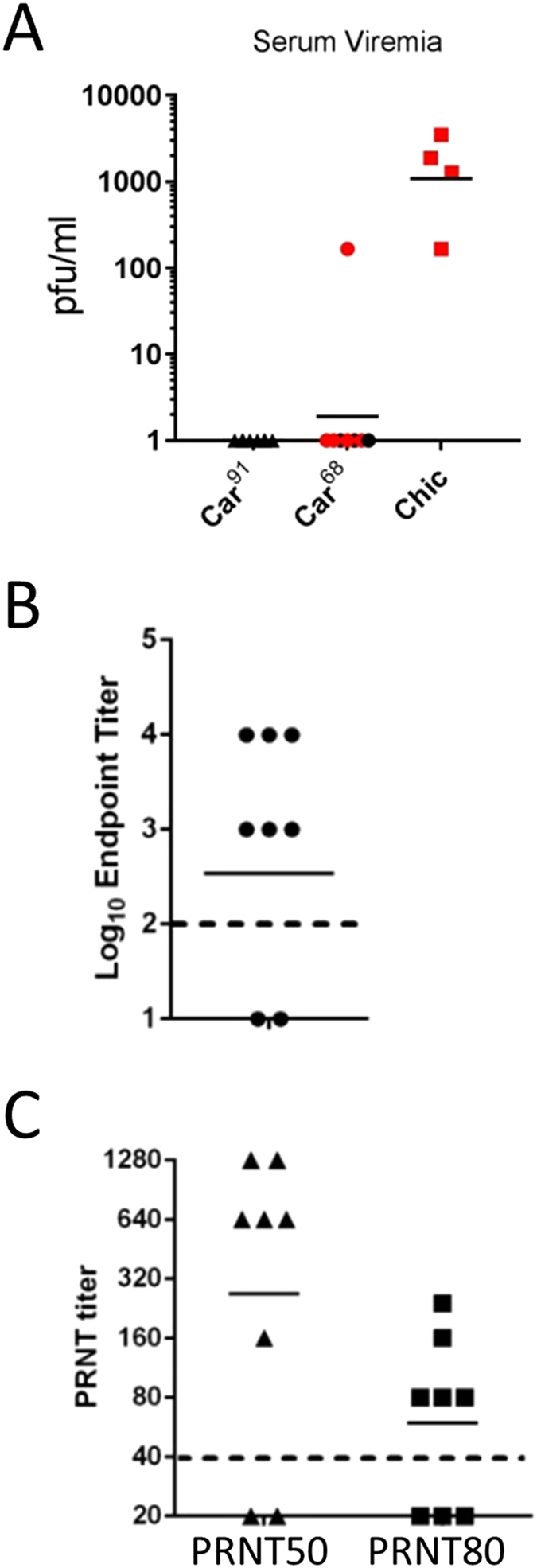
Evaluation of serum from MACV infected guinea pigs. (**A**) Viremia was determined on Vero cell monolayers using guinea pig serum taken from survivors on day 30 (black) or when animals were euthanized (red). The solid line represents the GMT values. (**B**) Antibody binding ELISA titers were determined by incubating sera from Car^91^-infected animals with PsVs pseudotyped with GPC from Strain Carvallo. Antiserum samples were serially diluted prior to incubation. The dashed line denotes the limit of detection. The solid line represents the GMT value. (**C**) Titer of neutralizing antibody in Car^91^-infected animals was determined and PRNT50 and PRNT80 titers were plotted. The dashed line denotes the limit of detection. The solid line represents the GMT values.

We previously reported^36^ that a MACV strain Carvallo variant (Car^91^) does not produce acute disease in Hartley guinea pigs (SFig. 1). Because the failure of Car^91^ to cause lethality was unexpected, we produced another stock of virus derived from an early passage of strain Carvallo (produced in 1968) and designated it Car^68^. The virulence of Car^68^ was examined in Hartley guinea pigs to determine if, contrary to Car^91^, this variant produced acute disease. As a control, one group of animals were infected with Chicava (Chic), a MACV strain known to cause lethal disease in this model^37^. Groups of eight animals were infected with the indicated strains and survival, weight and fever were monitored for 30 days (Fig. 1). All animals infected with strain Chic begin to lose weight between days 8–20 (Fig. 1B), but only one animal developed high fever ( > 41.0 °C) (Fig. 1C). All Chic-infected animals succumbed to infection by day 24. Animals infected with Car^68^ displayed weight loss between days 9–21, but none of the animals developed high fever ( > 41.0 °C). Car^68^ also produced a lethal disease in guinea pigs; however three animals survived infection (~63% mortality rate). Distinct from Chic, three Car^68^ infected animals developed paralysis starting with the hind-limb and were euthanized on day 21. The three surviving Car^68^ infected animals began to rapidly increase in weight after a period of weight loss, and by day 30 they exceeded their starting weight by ~3–20%. The mean time to death (MTD) for Car^68^ and Chic was 23.5 and 22 days, respectively. Confirming our earlier observations (SFig. 1), animals infected with Car^91^ survived infection without displaying signs of disease **(**Fig. 1A). Survival differences between Car^68^ and Chic infected animals were not significant (log-rank; p = 0.1331); however differences in survival between Car^91^ versus Car^68^ were highly significant (log-rank; p = 0.0082). Additionally, weight loss between Car^68^ and Chic were significant compared to Car^91^ for several days (Two-way ANOVA; p < 0.05). Viremia was detected in all four Chic-infected animals euthanized due to disease severity with GMT titers of 1,088 pfu/ml (Fig. 2A). Only one Car^68^-infected animal had detectable viremia (166 pfu/ml), and viremia was undetected in the Car^91^-infected group.

To gain insight into the interaction of Car^91^ and Car^68^ within infected guinea pigs, two additional groups of three animals each were infected with Car^68^ and Car^91^ as above and on day 14 viremia and hematology were evaluated. No viremia was detected for either strain. However, significant differences in white blood cells (WBC), lymphocyte numbers (LYMPH), and platelet (PLT) levels were observed between the avirulent and virulent Carvallo strains (SFig. 2). The avirulent strain had elevated WBCs, LYMPH and PLT values compared to animals infected with Car^68^ and uninfected control animals. The pathogenic Car^68^ strain had WBC and LYMPH levels equal to that of control animals, but reduced PLT values. Additionally, two of three Car^68^ infected guinea pigs had increased levels (but not statistically significant) of large unstained cells (LUCs), which is indicative of an acute viral infections^38^. Similar results were obtained for Chic (data not shown). Overall, our findings demonstrated that contrary to Car^91^, the Car^68^ strain variant can produce an acute and lethal disease in guinea pigs.

We next evaluated the serum from Car^91^-challenged guinea pigs for the presence of binding and neutralization antibodies 30 days post-challenge. ELISA titers were determined using VSVΔG particles pseudotyped with glycoproteins from the MACV strain Carvallo as antigen. Six of eight guinea pigs had detectable antibodies against MACV glycoprotein with a log_10_ GMT of 2.8 (Fig. 2B). MACV neutralizing antibody was detected in all but two infected animals with PRNT50 and PRNT80 GMTs of 269 and 59.5, respectively (Fig. 2C). The same two guinea pigs had no detectable PRNT50 or ELISA titers.

### Genomic analysis of Car^91^ and Car^68^

**Figure 3:**
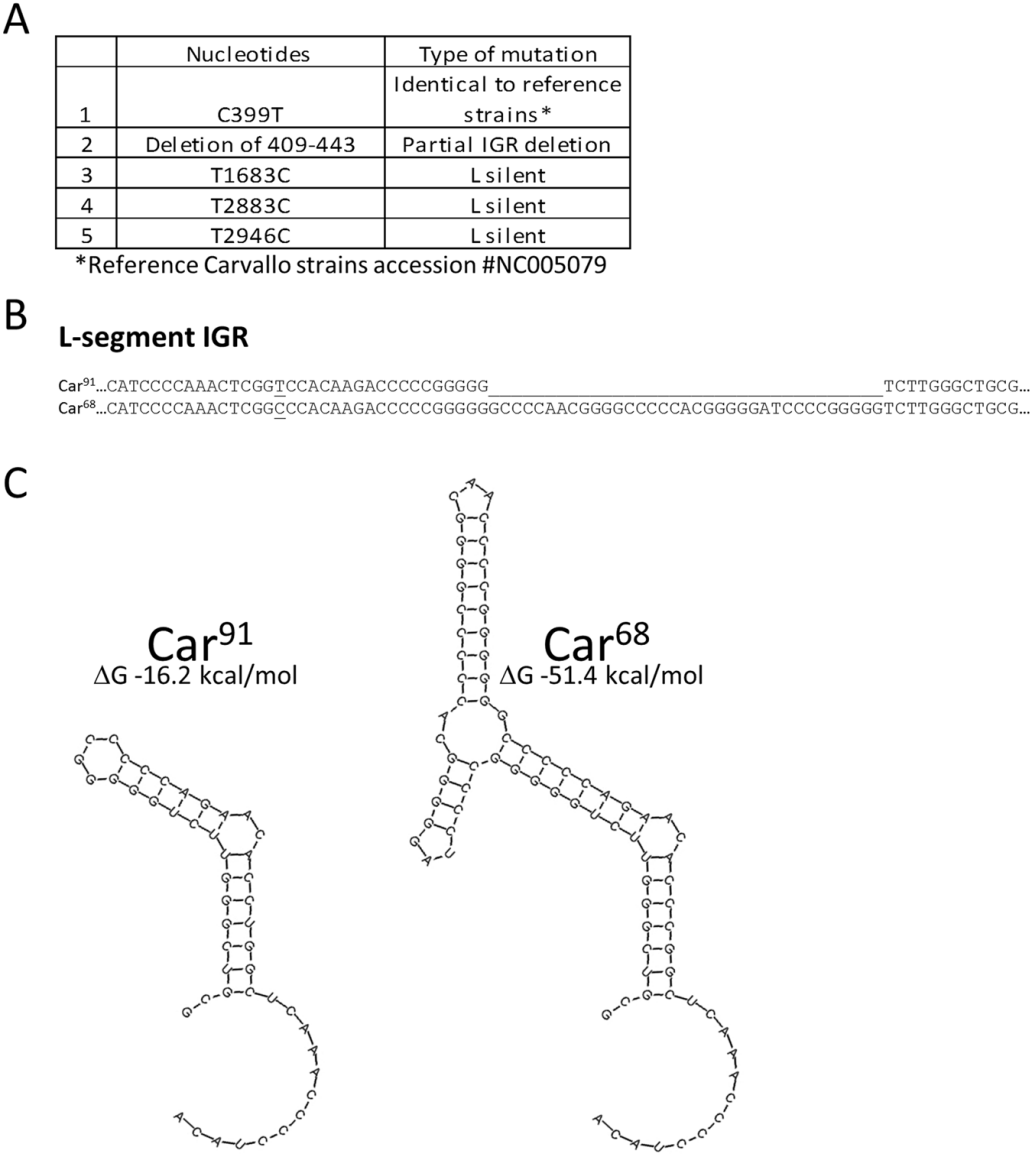
Sequence of MACV strain Car^91^ and Car^68^. (**A**) Predicted L-segment amino acid changes between Car^91^ and Car^68^. (**B**) Alignment of the Car^91^ and Car^68^ IGR. The underlined region denotes the region deleted in Car^91^. Also underlined is the single nucleotide change at position 399. (**C**) Predicted hairpin tertiary structure of the Car^91^ and Car^68^ L-segment IGR.

The genomes of Car^91^ and Car^68^ were sequenced to determine the genetic variation(s) that may contribute to virus attenuation. Sequencing revealed five changes between Car^68^ and Car^91^ in the L-segment (Fig. 3A). Three changes resulted in undisruptive silent nt substitutions in the polymerase protein sequence. Another nt change in the Car^91^ IGR at position 399 (C → U) was detected that matched the reference strain Carvallo sequence (Genbank accession #NC005079). We also identified a 35 nt deletion in the IGR of strain Car^91^ (Fig. 3B). No changes in the S segment were identified between Car^91^ and Car^68^.

Defective interfering (DI) particles are viral particles that carry mutations in the genome (typically deletions or rearrangements) that render the genome non-viable. During some infections, DI particles can accumulate to high levels through co-infection and disrupt the replication of viruses with viable genomes, leading to attenuation^39^. To examine whether a difference in the relative abundance of DI particles between Car^91^ and Car^68^ could be responsible for the attenuated phenotype of Car^91^, we examined the Illumina sequencing dataset in two ways. First, we looked for sequence coverage depth variation between the two isolates. Since DI particles often contain internal deletions, extreme levels of DI particles will skew sequencing coverage towards the ends of the genome fragments. Comparison of the patterns of sequence coverage depth between Car^68^ and Car^91^ did not reveal any obvious differences. For a more sensitive assay, we looked for evidence of chimeric reads, which would also be indicative of DI particle abundance. Chimeric reads are reads that span a deletion or rearrangement breakpoint, resulting in the 5′ and 3′ ends of the read aligning to non-adjacent regions of a reference genome. We detected 0.66% chimeric reads for Car^68^ and 0.43% chimeric reads for Car^91^. Additionally, the pattern of deletions across the genome segments (by size and location) is similar between the two viruses (SFig. 3). Since the proportion of chimeric reads is higher in Car^68^, indicating that Car^68^ may actually have a higher proportion of DI particles, and the pattern of deletions across the genome is qualitatively similar, it is unlikely that DI particles are contributing to the attenuated phenotype of Car^91^.

Altogether, these findings demonstrated that Car^91^ has a significantly altered L-segment IGR relative to the earlier passaged Car^68^, including a 35 nt partial deletion. This disruption resulted in a predicted IGR structure with a ΔG value of −16.2 kcal/mol compared to −51.4 kcal/mol of Car^68^ (Fig. 3C). Thus, the Car^68^ structure is predicted to be more thermodynamically stable compared to Car^91^ by 3.2-fold.

### ***In vitro*** characterization of Car^91^, Car^68^ and Chic

**Figure 4:**
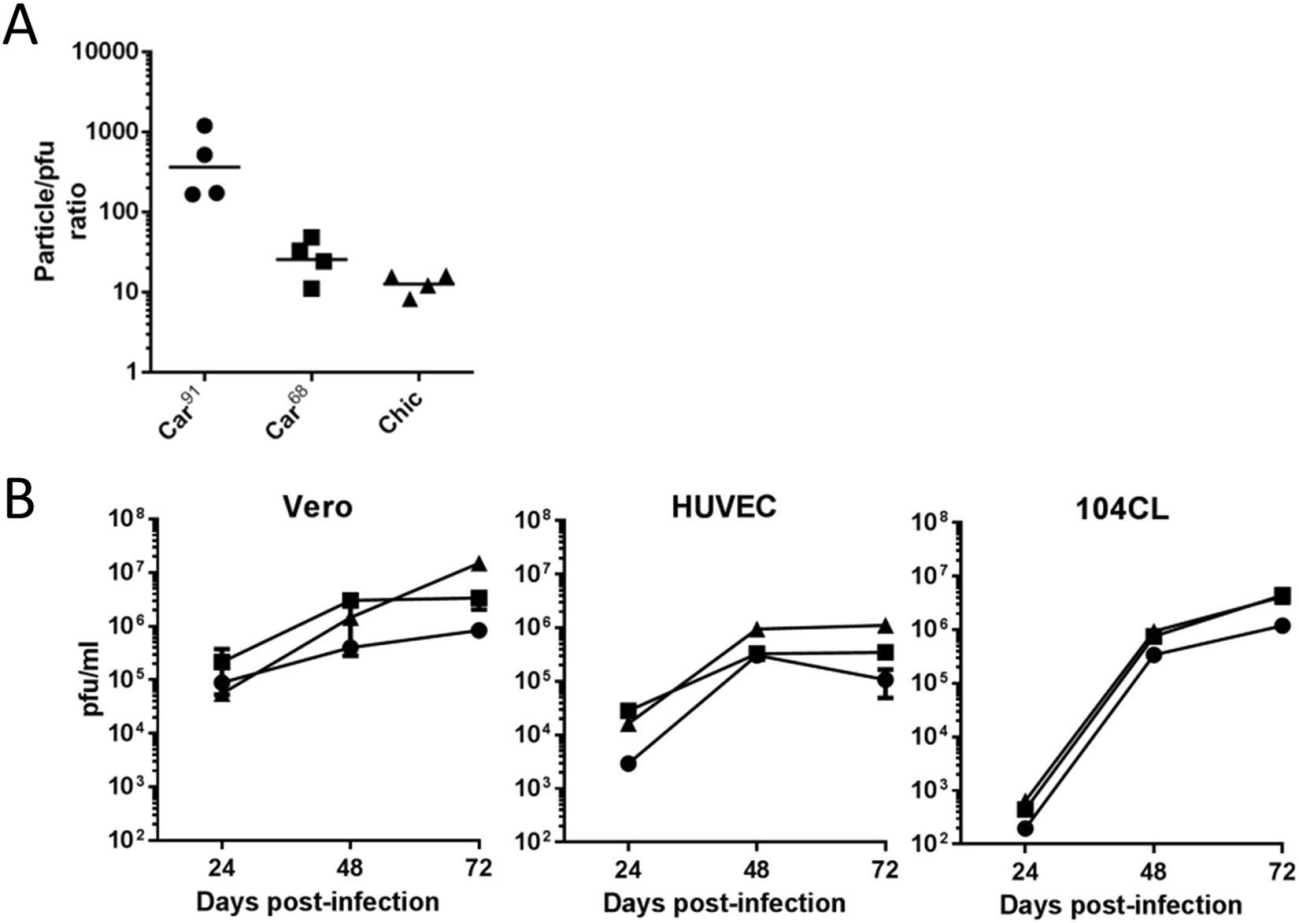
*In vitro* characterization of Car^91^, Car^68^ and Chic. (**A**) Particle-to-pfu ratios for each of the indicated MACV strains were determined using a ViroCyt system. Four virus preparations for each virus were tested in triplicate. The geometric means are indicated by the solid line. (**B**) The indicated cell types were infected with Car^91^ (circles), Car^68^ (squares) or Chic (triangles) at an MOI of 0.1 and replication was quantitated at 24, 48 and 72 hpi by plaque assay. All samples were titered in duplicate and the mean + /− SD were graphed.

To begin to address the basis of the attenuation of Car^91^, we examined both Carvallo variants Car^91^ and Car^68^, and strain Chic for differences in particle-to-pfu ratios and replication kinetics. Car^91^ had the highest particle-to-pfu ratio with a geometric mean of 369, compared to Car^68^ and Chic whose GMT ratios were 26 and 13, respectively (Fig. 4A). These findings indicate that compared to Car^68^, Car^91^ has a ~14-fold increase in the particle to pfu ratio. However, these differences were not statistically significant (T-test; p > 0.05).

The growth kinetics of both strain Carvallo variants and strain Chic were investigated in Vero cells, 104CL guinea pig fibroblasts and Human umbilical vein cells (HUVECs). Cells were infected with Car^91^, Car^68^ or Chic and replication was assayed at 24, 48 and 72 h post-infection (hpi) (Fig. 4B). After 24 h, Car^68^ grew to the highest levels in both Vero and HUVEC. 24 h growth for all three viruses in 104CL cells was markedly lower than Vero and HUVECs, however Car^91^ titers were the lowest in these cells. At 48 hpi, Car^91^ replication was still reduced compared to the other viruses in 104Cl and Vero cells, however in HUVECs Car^91^ and Car^68^ had similar titers. After 72 h, Chic had the highest titers in Vero and HUVECS. Titers of Car^68^ and Chic were similar in 104CL cells at this time point. Overall, Car^91^ replicated the poorest in all cell types tested with titers several fold lower than those of Car^68^ and Chic. The replication differences between Car^91^ and Car^68^ were statistically significant (two-way ANOVA; p < 0.05) at 72 h in HUVECs and 104CL cells, but not in Vero cells. Growth titers were also significantly different between Car^91^ and Chic at 72 hpi in all cell types (two-way ANOVA; p < 0.05). Together these findings indicated that Car^91^ does not replicate as efficiently in cell culture as the virulent Car^68^ and Chic strains.

### MACV strain Car^91^ protects guinea pigs against lethal infection by GTOV

**Figure 5:**
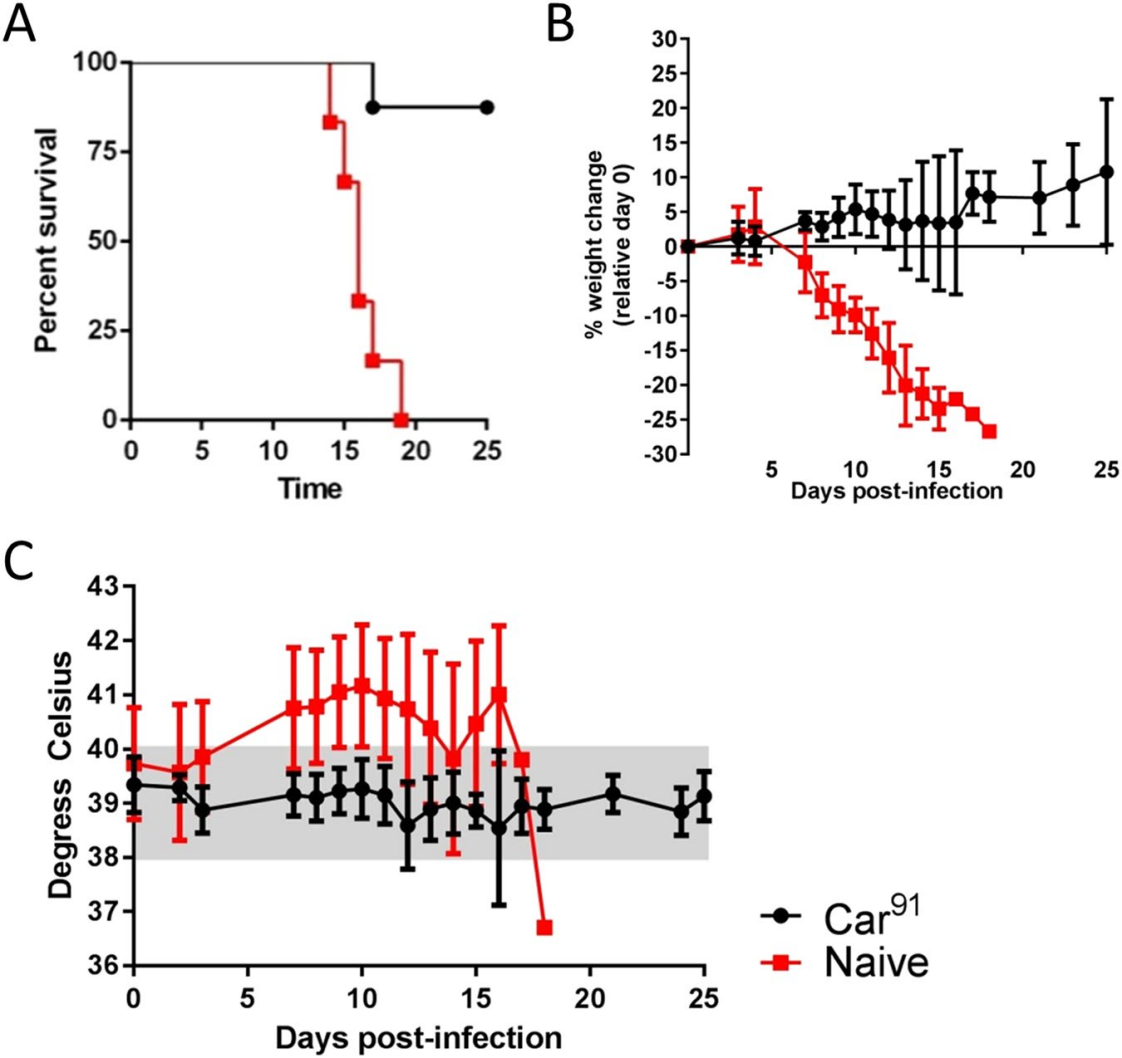
Protective efficacy of MACV strain Car^91^ against lethal GTOV challenge in guinea pigs. (**A**) Survival plot of naïve (red squares) and Car^91^-vaccinated (black circles) guinea pigs infected by the i.p. route with 2,000 pfu GTOV. Survival was plotted for 30 day post-infection. (**B**) Percent loss from starting weight was plotted for each group as described above. (**C**) Temperature was monitored as in Fig. 3.

Because Car^91^ was highly attenuated in guinea pigs but produced detectable immune responses in 10/12 guinea pigs (SFig. 1 and Fig. 4), we hypothesized that it might function as an attenuated vaccine. Therefore, we examined the ability of Car^91^ to protect guinea pigs against GTOV, a distantly related human pathogenic NW arenavirus species and causative agent of Venezuelan hemorrhagic fever^34, 40, 41^. Eight guinea pigs were challenged with GTOV 45 days after exposure to Car^91^ (Fig. 5). As a control for acute infection, a group of six weight-matched naïve guinea pigs were also infected with GTOV. Animals were monitored for survival, weight loss and fever over 25 days (Fig. 5A–C). Consistent with previous findings^36, 41^, control animals began to lose weight starting around day 6 with concomitant fever. All control animals succumbed to infection with a MTD of 16 days. All but one animal previously exposed to Car^91^ survived infection. The single non-survivor succumbed to disease on day 17 after a period of weight loss and mild fever (~40.3 °C).

**Figure 6:**
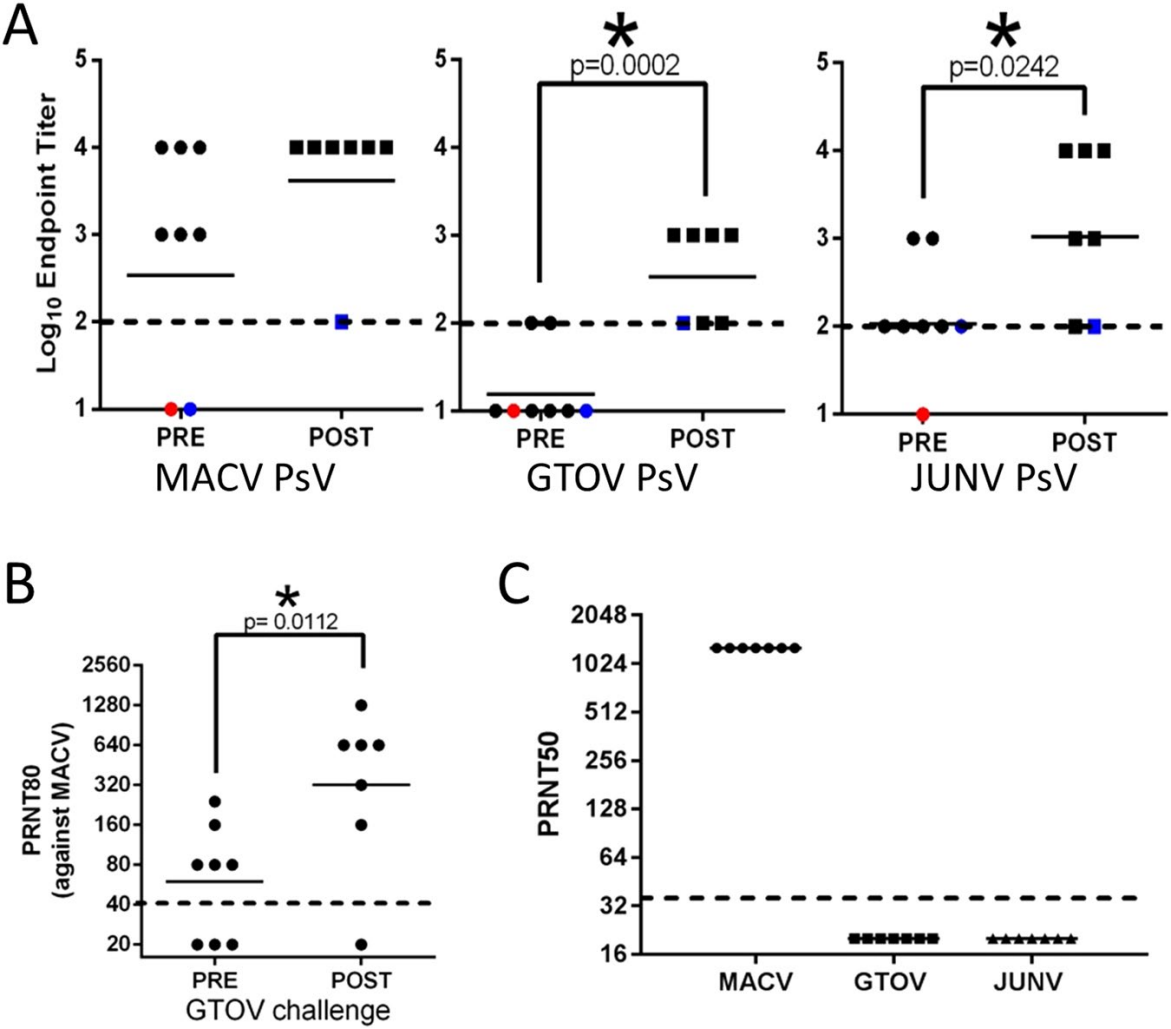
Binding and neutralizing antibody responses in guinea pigs infected with GTOV. (**A**) Antibody binding titers were determined by coating 96-well plates with the indicated PsVs and incubating them with serially diluted antiserum samples from before (circles/PRE) or after (squares/POST) challenge with GTOV. The dashed line denotes the limit of detection. The red circle denotes the single animal (Animal #4) that succumbed to infection. The blue symbols denote the same animal before and after GTOV challenge. Note that titers against MACV prior to GTOV challenge are also depicted in Fig. 4B. (**B**) PRNT80 titers against MACV (Car^68^) prior to and after challenge with GTOV were determined as above. Note that PRNT80 titers prior to GTOV challenge are also depicted in Fig. 4C. (**C**) PRNT50 titers against JUNV, MACV and GTOV were determined as in Fig. 2. Titers were determined as described above. The dashed line indicates the limit of detection. For all panels, asterisks denote statistical significance.

The presence of binding antibody against MACV, JUNV and GTOV was evaluated by ELISA using sera from Car^91^-exposed (vaccinated) guinea pigs collected prior to and 30 days after GTOV challenge (Fig. 6A). Prior to GTOV challenge, six of eight animals infected with MACV strain Car^91^ had detectable antibodies against MACV with a log_10_ GMT of 2.5. These responses increased following GTOV challenge to 3.6, but this increase was not significant (T-test; p = 0.1461). Antibody titers against GTOV prior to GTOV challenge were low or below detection. However, antibody titers against GTOV rose significantly (T-test; p = 0.0002) after GTOV challenge to a log_10_ GMT of 2.5. Antibody titers against JUNV were also detected prior to GTOV challenge (log_10_ GMT 2.0) in all but one animal and these responses significantly increased after GTOV challenge (T-test; p = 0.0242) with a log_10_ GMT of 3.0. Animal#4, which succumbed to GTOV infection despite receiving Car^91^, had undetectable ELISA titers against MACV, GTOV and JUNV and an undetectable PRNT50 titer against MACV. Animal#1 survived GTOV challenge despite having no detectable humoral responses against GTOV and MACV, and a low response against JUNV ELISA antigen.

The PRNT titers against MACV significantly increased subsequent to GTOV challenge, with GMT PRNT80 titers rising from 59 to 320 (T-test; p = 0.0112) (Fig. 6B). Despite the presence of MACV, GTOV and JUNV IgG antibody in all animals surviving GTOV challenge, we did not detect any neutralizing activity against GTOV or JUNV (Fig. 6C). These findings demonstrated that guinea pigs inoculated with Car^91^ are protected against heterologous challenge by GTOV. However, this protection occurred in the absence of detectable neutralizing antibody responses against the challenge virus.

## Discussion

A major goal of arenavirus vaccine design is to develop a pan-arenavirus vaccine that protects against heterologous species within either the OW and NW complexes, or more broadly. Previous work has shown that JUNV, MACV and TACV can cross-protect against each other in animal models^24, 28^. However, we and others have found that JUNV and MACV to be much more serologically related (based on cross-neutralization and GP1 cross-binding) compared to GTOV^36^. Accordingly, to thoroughly gauge the level of heterologous protection against other NW arenaviruses, we purposely challenged Car^91^-vaccinated guinea pigs with the more genetically and serologically distant GTOV^36, 40^. We also delayed challenge for 45 days after the initial inoculation with Car^91^ to avoid any transient innate immune effects that may have enhanced protection. The single animal succumbing to GTOV challenge failed to produce detectable antibodies against even MACV. We predict a higher dose (>10-fold) of Car^91^ would have elicited more robust and protective immune responses and thus resulted in 100% protection. This prediction is based on the fact that the dose of the JUNV attenuated vaccine Candid#1 in humans is 40,000 pfu^20^, whereas here we used Car^91^ at 1,000 pfu. Curiously, neutralizing antibody against GTOV was not detected in any animal even after GTOV challenge. Other studies demonstrate that TACV protects against JUNV infection in the absence of neutralizing antibody responses targeting the challenge virus^28^. These data support a model whereby non-neutralizing antibody and/or cytotoxic T-cell responses may play essential roles in protection against heterologous challenges. More work will be needed to fully address the correlates of protection however; our findings clearly indicate that attenuated MACV strains can produce cross-protective immune responses against distantly related arenaviruses at least within the same complex. While not tested in our study, it is likely Car^91^-inoculated animals would have been protected against challenge by a virulent strain of MACV (i.e. Car^68^ or Chic). JUNV strain candid#1 protects against JUNV in infected animals, and this correlates with neutralizing antibody responses^21^.

We previously reported that treatment of guinea pigs with anti-MACV neutralizing antibodies significantly reduces the humoral immune responses against Car^91^, suggesting the avirulent isolate had to replicate to some extent within infected animals to produce adequate immune responses^36^. This is supported by hematology data (SFig. 2) showing that the numbers of WBC/LEUKO are elevated over control animals two weeks after inoculation with Car^91^ indicating an active immune response against the avirulent strain. Interestingly, WBC/LEUKO values after Car^68^ (SFig. 2) and Chic (data not shown) exposure were similar to the controls, which is consistent with the ability of virulent strains of arenaviruses to cause suppression of the leukocyte responses^34^. Similarly, PLT values were also decreased for virulent strains consistent with the ability of arenaviruses to cause thrombocytopenia in infected hosts^34^. Importantly, Car^91^ did not cause any apparent signs of disease such as weight loss or fever and LUC values of exposed animals, which are indicative of acute viral infections^38^, were not elevated, but were for the virulent Car^68^ isolate.

Arenavirus IGRs are situated between each encoded ORF on both L and S segments^8^, and play important roles in transcription and production of infectious progeny virions^42^. Mechanistically, IGRs fold into single or double stem-loop structures and are essential for transcription termination. Because the tertiary structure of the IGR is critical for mRNA transcription termination, modifications can significantly impact the efficiency of replication by disrupting protein synthesis. For example, truncation of the LUJV L-segment IGR produces a virus that replicates less efficiently *in vitro* due to inefficient gene transcription^43^. Our findings strongly suggest that spontaneous alteration of the L-segment IGR is chiefly responsible for the loss of virulence of the Car^91^ variant in guinea pigs. High levels of DI particles in the Car^91^ stock could also potentially cause attenuation, but examination of the sequencing data does not support the presence of higher levels of DI particles in the Car^91^ stock compared to the Car^68^ stock. The three silent mutations detected in the L protein open reading frame do not impact amino acid coding, making it unlikely they could contribute largely to attenuation. Thus, the only substantial difference between the avirulent Car^91^ and virulent Car^68^ is the L-segment IGRs. However, future studies using available MACV reverse genetic systems^44^ will be needed to fully determine if the 35 nt partial IGR deletion alone is solely responsible for the attenuation.

It is unclear how a partial L-segment IGR deletion arose during passage of strain Carvallo. The available evidence indicates that Car^91^ was passaged two additional times in VeroE6 cells compared to Car^68^. Some work has shown that MACV can be attenuated by cell culture passage^23^, but these studies did not report if attenuation involved IGR modification. Curiously Yun, *et al*., reported that the JUNV strain XJ was apathogenic in guinea pigs after additional passaging in mouse brains^45^. Other variants of XJ are known to be virulent in the guinea pig model^46–48^. It was not reported if this attenuation was the result of an IGR mutation or some other factor(s). The spontaneous loss of virulence of different mammalian arenaviruses as a result of propagation underscores the need to obtain sequencing data from clinical isolates as soon as they emerge to prevent the deleterious effects of cell culture or animal adaptation of the viral genome.

Car^91^ had reduced replication fitness *in vitro* compared to Car^68^, including reduced replication in primary endothelial cells, which are *in vivo* targets of the virus^49^. However, these growth defects were relatively modest. Notably, the 1.5 log reduction in growth was similar to that observed for the LUJV containing the partial L-segment IGR deletion^43^. Whether the Car^91^ replication deficiency alone results in the attenuation *in vivo* is not clear. It is possible that reduction in the size of the IGR and the resultant bearing on its tertiary structure may impact innate immune signaling pathways within infected cells, and this could play a critical role in attenuation. Hyperstimulation of the innate immune response as evidenced by interferon (IFN) stimulated gene expression has been observed in cell culture for attenuated JUNV vaccine strain Candid#1^50^. Future studies will be needed to fully elucidate the mechanism(s) by which partial IGR deletion produces apathogenic arenaviruses *in vivo*, with particular emphasis on activation of IFN stimulated gene products. Such analysis will benefit from the development of transcriptomic platforms specific to guinea pig gene expression^51^.

Recent work by Iwasaki, M. *et al*. has focused on exploiting alterations in the IGR as a means of producing rational whole-virus vaccines against arenaviruses. Addition of synthetic (non-viral S-IGR like) sequence or swapping the IGRs of the L- and S-segments produces attenuated viruses that can protect mice against secondary challenge with wild-type LCMV^52, 53^. Our work indicates that modification of the L-segment IGR, including a 35 nt deletion, can also produce an attenuated virus that functions as a vaccine. Whether substitution of the IGR, incorporation of a synthetic IGR or deletion of the IGR is the best approach in live-attenuated arenavirus vaccine development remains to be determined. One advantage to deletion of multiple nucleotides is that reversion to wild-type is improbable. Work involving the arenavirus IGR as a vaccine strategy has focused exclusively on OW arenaviruses, specifically LCMV. Our study advances these vaccine strategies by supporting alteration of the IGRs as a powerful means of producing an attenuated live-virus vaccine against NW arenaviruses.

Many human pathogenic arenaviruses are endemic and well-described, such as LASV which causes >100,000 infections annually, in addition to several of the South American arenaviruses including JUNV, MACV and GTOV^54^. However, novel human pathogenic arenaviruses emerge at unpredictable rates in both the Americas and Africa^2, 13–16, 55^, the most recent being Lujo virus in Africa. Accordingly, any vaccine-based countermeasure should be designed to protect broadly protect against known and unknown arenaviruses. Our work and the work of others^52, 53^ support the use of IGR-modification as a strategy for pan-arenavirus vaccine development. With the advent of arenavirus reverse genetics^56^, other attenuation strategies such as codon deoptimization^57, 58^ could be combined with IGR-modification to produce rationally-designed vaccines that are both highly-attenuated, yet replication competent and safe for human use.

## Methods

### Viruses and cells

GTOV strain INH95551, MACV strain Chicava and two MACV strain variants of Carvallo from passages dated 1968 (Car^68^) and 1991 (Car^91^) were propagated as previously reported^36^. All viruses were twice plaque purified prior to use. Car^68^ was passaged twice in sucking hamster brains and once in VeroE6 cells. Car^91^ was passaged an addition two times in VeroE6 cells. 239 T cells and 104CL guinea pig fibroblasts (ATCC) were maintained in MEM or RPMI containing 10% heat-inactivated fetal bovine serum (FBS), 1% antibiotics (100 U/ml penicillin, 100 μg/ml of streptomycin, respectively. HUVECs were purchased from a commercial source (Lonza) and maintained in endothelial growth medium.

### Challenge of Hartley guinea pigs

Female Hartley guinea pigs (300–400 g) were implanted with IPTT-3000 identification chips to monitor temperature (BMDS INC; Seaford, DE). Animals were challenged with the indicated MACV strains (1,000 pfu) or GTOV (2,000 pfu) diluted in a total volume of 0.5 ml PBS by intraperitoneal (i.p.) injection. Animals were weighed and monitored for fever. All animal studies were conducted in compliance with the Animal Welfare Act and other federal statutes and regulations relating to animals and experiments involving animals and adheres to principles state in the *Guide for the Care and Use of Laboratory Animals*, National Research Council^59^. All animal experimental protocols were approved by a standing internal institutional animal care and use committee (IACUC). The facilities where this research was conducted are fully accredited by the Association for Assessment and Accreditation of Laboratory Animal Care International. Animals meeting criteria were humanly euthanized.

### Plaque reduction and neutralization tests (PRNTs)

PRNTs were performed as previously described^60^ using guinea pig serum serially diluted two-fold starting at 1:40. Percent neutralization was calculated relative to the number of plaques in the presence of negative control serum. Titers represent the reciprocal of the highest dilution resulting in a 50% reduction in the number of plaques. Data were plotted using Graphpad Prism software.

### Growth kinetics

Vero, 104CL and HUVECs were seeded at a density of 1 eql × 10^5^ cells per well in 24-well plates and infected at an MOI of 0.1 with the indicated viruses diluted in culture medium. Virus growth at 24, 48 and 72 h was determined by plaque assay on Vero cell monolayers. All samples were run in two independent replicates and plotted as the mean + / − standard deviation (SD) using Graphpad Prism software.

### Particle-to-PFU ratio

Particle counts were determined with a Virocyt machine (Virocyt, Boulder, CO) using the manufacture’s protocol. The particle-to-pfu ratios were determined by dividing particle counts by the amount of infectious virus. Four independent virus preparations per strain were used in the calculations.

### Genome sequencing

RNA was extracted from Trizol homogenates of MACV, converted to cDNA, and subjected to sequence-independent, single primer amplification (SISPA)^61^. The products of these reactions were used to generate libraries that were sequenced on an Illumina MiSeq. Sequencing reads were assembled using DNAStar SeqMan NGen. Predicted secondary structures of the IGRs were determined using DNAstar Genequest.

### Identification of defective genomes

To look for putatively defective vNiral genomes (i.e., genomes with large deletions or rearrangements), we identified chimeric reads from the Illumina dataset. Chimeric reads were defined as reads with 1) two distinct, non-overlapping alignments to different regions of the MACV genome and with 2) ≥99% of read bases aligned to the reference when considering both alignments together. To prevent bleed through between multiplexed samples, we used non-overlapping dual indexes and we filtered out any reads with index base qualities less than Phred 20, on average. Only the first read from each pair was used to avoid double counting. Illumina and SISPA adaptors were clipped using Cutadapt v1.9.dev1^62^ and Prinseq-lite v0.20.3^63^ was used to 1) remove 6 nt from the beginning and end of each read (“*–trim_right 6 –trim_left 6*”; to remove random hexamers), 2) trim low quality bases from the 3′ ends of the reads (“*–trim_qual_right 30 –trim_qual_type min –trim_qual_window 5*”), 3) remove reads <40nt in length or with a mean quality score less than Phred 20 (“*–min_len 40 –min_qual_mean 20*”), 4) remove reads with low complexity (“*–lc_method dust –lc_threshold 3*”) and 5) remove exact duplicates (“*–derep 14*”). Reads were then aligned to a reference sequence (Car^68^ GenBank: KM198592.1 and KM198593.1; Car^91^ sequences have been uploaded to GenBank) using BWA mem v.0.7.12 with default parameters^64^. Chimeric reads were identified and characterized using a custom script, chimeric_reads.py v3.5.4 (https://github.com/jtladner/Scripts/tree/master/chimeric_reads). Note that the S-segment consensus sequence for Car^91^ exactly matches that of Car^68^ (KM198592.1).

### Pseudovirion neutralization assay (PsVNA) and ELISA

The pseudovirion neutralization assay (PsVNA) has been described in detail elsewhere^36, 65^. Briefly, a vesicular stomatitis virus backbone with a luciferase reporter gene (PsV) was used to produce particles decorated with glycoproteins from MACV, JUNV and GTOV. These particles were subsequently incubated with the indicated serially diluted sera in triplicate and the geometric mean PsVNA80 titers (GMT) plotted. The use of PsV as solid phase antigen in ELISAs has been previously described in detail^36^.

### Statistical analysis

Two-way ANOVA with the Bonferroni correction was used to analyze both weight and viral replication (*in vitro*). Log-rank test was performed for statistical analysis of survival. The statistical significance of PRNTs was determined using an unpaired two-tailed Student’s *t* test. Significance levels were set at a *p* value less than 0.05. All analyses were performed using Prism software.

### Data Availability

All data generated or analyzed during this study are included in this published article (and its Supplementary Information files) with the exception of the genomic data for Car^91^. The sequencing information for Car^91^ has been uploaded to Genbank.

## Electronic supplementary material

Supplementary Information
