# Supplementary material for: An attenuated Machupo virus with a disrupted L-segment intergenic region protects guinea pigs against lethal Guanarito virus infection
Source: Sci Rep. 2017 Jul 5;7:4679. doi: 10.1038/s41598-017-04889-x (PMC5498534; doi:10.1038/s41598-017-04889-x)
Supplement: Supplementary file 1 — Supplementary Information [file 41598_2017_4889_MOESM1_ESM.pdf]

An attenuated Machupo virus with a disrupted L-segment intergenic region protects guinea pigs against lethal Guanarito virus infection

Joseph W. Golden[1*], Brett Beitzel[2], Jason T. Ladner[2], Eric M. Mucker[1], Steven A. Kwilas[1], Gustavo Palacios[2] and Jay W. Hooper[1]

Supplemental material

MACV strain Carvallo is not lethal in Hartley guinea pigs. MACV strain Car[91] was tested for its ability to produce acute disease in Hartley guinea pigs. Six guinea pigs were infected with 2,000 pfu of Car[91]and monitored for weight (SFig. 1A) and temperature (SFig. 1B) for 28 days post infection. All animals gained weight over the course of the study and did not exhibit signs of infection, such as lethargy or fever. Serum from infected animals was tested for neutralizing antibody against MACV using VSV particles pseudotyped with the MACV strain Carvallo glycoproteins. Each guinea pig produced a neutralizing antibody response with a PsVNA80 geometric mean titer (GMT) of 177 (SFig. 1C). These results indicated that the Car[91] Carvallo variant failed to produce acute disease in infected guinea pigs. However, the virus was able to elicit neutralizing antibody responses in inoculated animals.

Supplemental Figure 1. Infection of Hartley guinea pigs with Car[91]. A. Guinea pigs were infected with 2,000 pfu MACV strain Car[91] and weights based on day 0 starting weight were graphed. B. Percent change in temperature relative to day 0 was plotted. C. Serum from guinea pigs was incubated with VSVΔG particles pseudotyped with MACV strain Carvallo glycoproteins and the PsVNA80 titers were graphed.

Hematological analysis of Car^{91} and Car^{68} infected guinea pigs. Two groups of three guinea pigs per group were infected with MACV strain Car^{91} or Car^{68} and a third group of two animals was left uninfected to serve as controls. On day 14 post-infection, hematological analyses were conducted on all guinea pigs using whole blood using an ADVIA hematology analyzer. These results are described in the main manuscript.

Supplemental Figure 2. Hematology on Car^{91}, Car^{68} and uninfected guinea pigs. A. Guinea pigs were infected with 1,000 pfu MACV strain Car^{91} or Car^{68}. On day 14 post-infection, PBMCs were taken and hematology analyzed (ADIVA). Hematological values for WBC, PLT, LYMPH and LUC were graphed. Asterisks denote statistical significance (t-test; $p<0.05$).

Analysis for the presence of Defective Interfering particles. To examine whether a difference in the relative abundance of DI particles between Car^{91} and Car^{68} could be responsible for the attenuated phenotype of Car^{91}, we examined the Illumina sequencing dataset in two ways. These results are described in the main manuscript.

Supplemental Figure 3. Distribution of genomic deletions inferred from individual, chimeric Illumina reads. (A, B) S segments and (C, D) L segments for MACV Car^{68} and Car^{91}, respectively. Each circle represents a unique deletion. The size of each circle is relative to the number of distinct supporting reads and the placement indicates the location within the genome. The y-axis indicates the position of the first deleted nucleotide and the x-axis indicates the position of the last deleted nucleotide. The further a circle is from the dashed diagonal line, the

longer the deletion. Each circle is colored according to the relative density of circles (i.e., unique deletions) within that portion of the plot.

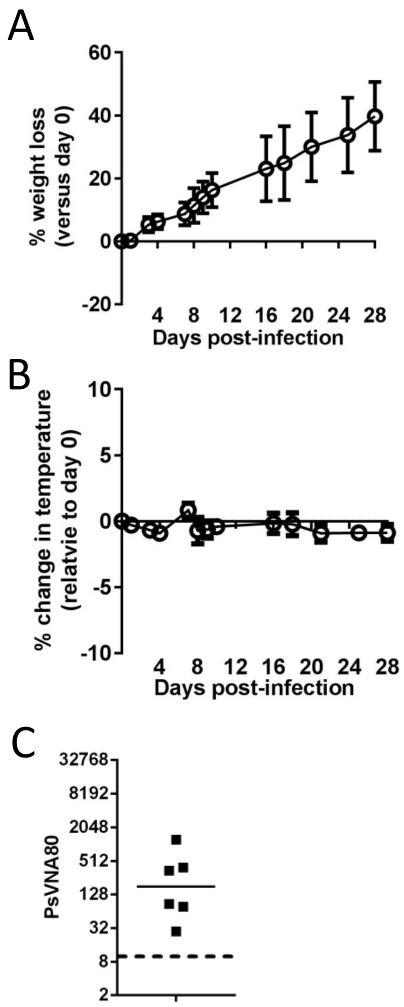
A
60
40
20
0
-20
% weight loss
(versus day 0)
4 8 12 16 20 24 28
Days post-infection
B
10
5
0
-5
-10
% change in temperature
(relatvie to day 0)
4 8 12 16 20 24 28
Days post-infection
C
32768
8192
2048
512
128
32
8
2
PsVNA80

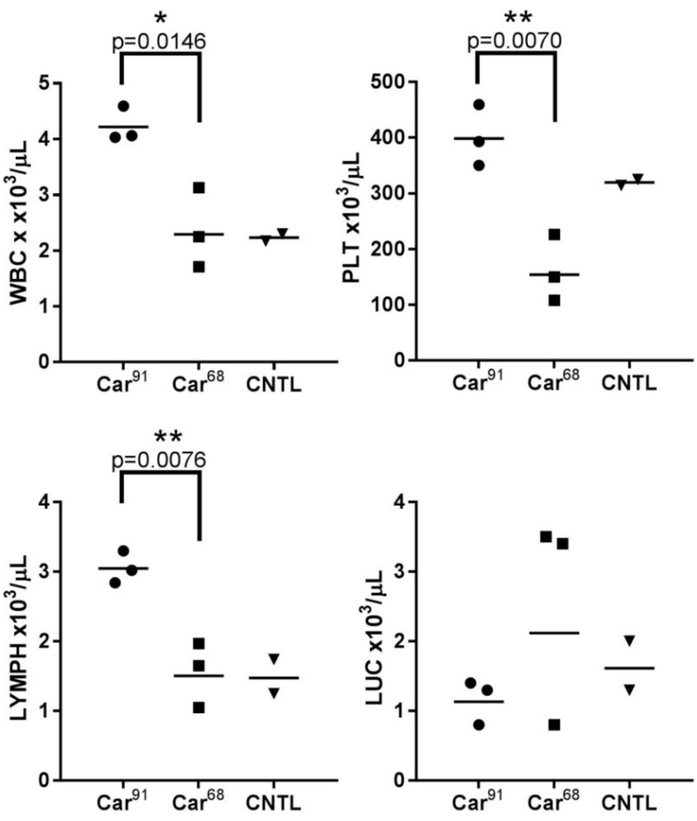
*
p=0.0146
WBC x x10³/µL
Car91
Car68
CNTL
**
p=0.0070
PLT x10³/µL
Car91
Car68
CNTL
**
p=0.0076
LYMPH x10³/µL
Car91
Car68
CNTL
LUC x10³/µL
Car91
Car68
CNTL

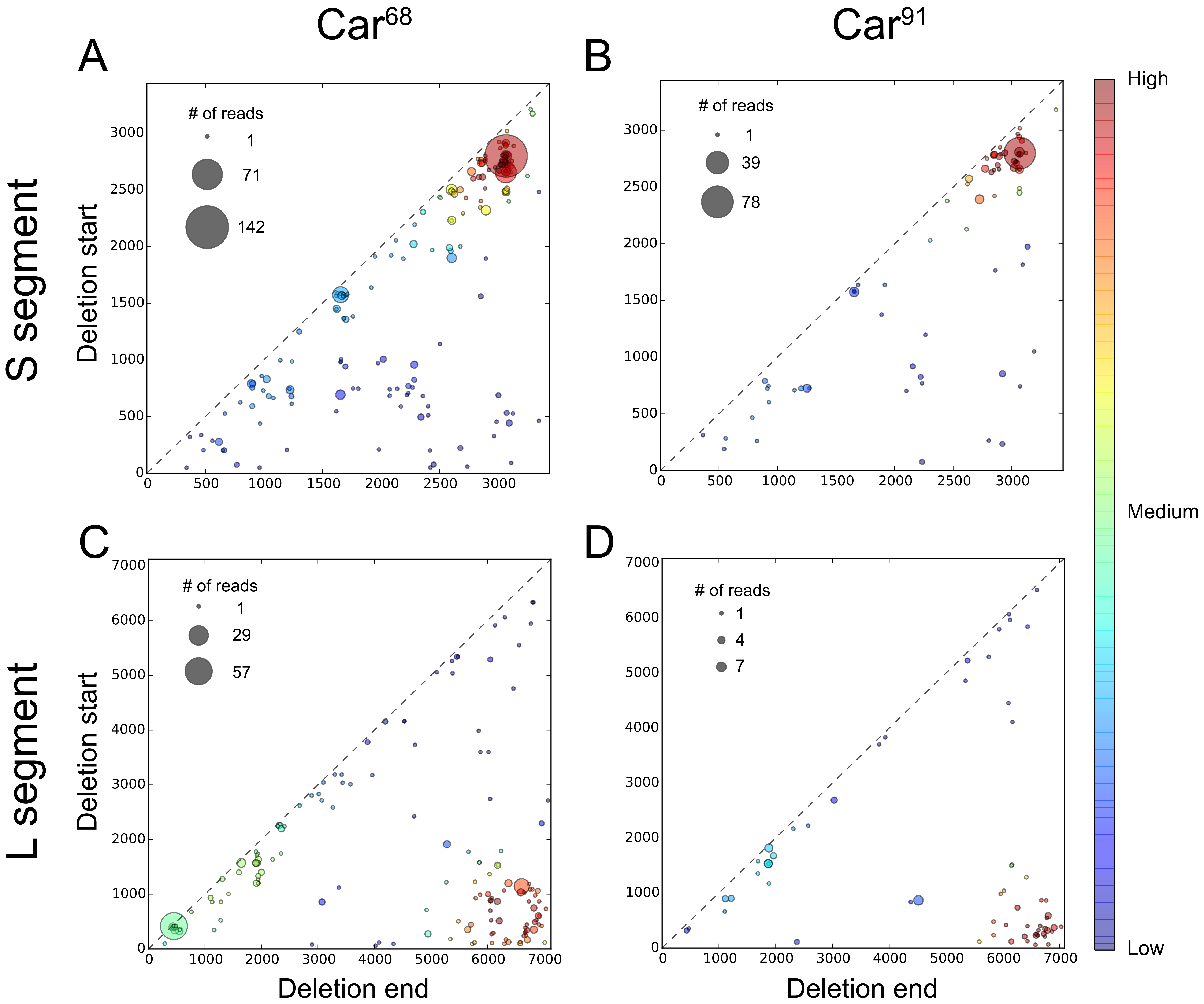

Car68
Car91
A
B
C
D
S segment
L segment
Deletion start
Deletion end
# of reads
1
71
142
1
39
78
1
29
57
1
4
7
High
Medium
Low
